# Sleep efficiency is negatively associated with resting daytime heart rate in trauma‐exposed premenopausal women

**DOI:** 10.14814/phy2.70525

**Published:** 2025-08-19

**Authors:** Chowdhury Tasnova Tahsin, Redeat Wattero, Zynab Ahmed, Chowdhury Ibtida Tahmin, Jennifer Esala, Donald L. Bliwise, Ida T. Fonkoue

**Affiliations:** ^1^ Divisions of Physical Therapy and Rehabilitation Science, Department of Family Medicine and Community Health University of Minnesota Medical School Minneapolis Minnesota USA; ^2^ Evaluation & Research Center for Victims of Torture Saint Paul Minnesota USA; ^3^ Department of Neurology Emory University Medical Center Atlanta Georgia USA

**Keywords:** blood pressure, heart rate, premenopausal women, sleep, trauma

## Abstract

Poor sleep is associated with increased cardiovascular events and mortality. Women are typically more exposed to interpersonal trauma than men and may be vulnerable to sleep disturbances as sequelae of trauma. Yet, the association between poor sleep and cardiovascular biomarkers such as resting blood pressure (BP) and heart rate (HR) in young trauma‐exposed women has not been well elucidated. We hypothesized that sleep quality would be associated with resting daytime BP and HR, independently of other factors. We recruited 131 otherwise healthy trauma‐exposed premenopausal women and measured resting daytime BP and HR using automated vital signs monitor and peripheral arterial tonometry. Participants wore wrist actigraphy for 7 days to assess objective sleep along with a sleep diary and completed two sleep questionnaires: the Pittsburgh Sleep Quality Index (PSQI) and the Insomnia Severity Index (ISI). Our analyses revealed that objective sleep efficiency was negatively correlated with (*r* = −0.222, *p* = 0.010) and was a significant predictor of (*β* = −0.206, *p* = 0.008) resting supine HR, even after controlling for age, BMI, BP, PCL‐5, and medication use for depression, anxiety, and sleep (*R* ^2^ = 0.253, *p* = 0.008). There was a weak correlation between ISI and diastolic BP (*r* = 0.192, *p* = 0.026). These results underscore the potential role of sleep in mental health‐associated cardiovascular disease.

## 1 INTRODUCTION

Cardiovascular disease (CVD) is the leading cause of death globally, making up 32% of all deaths in 2020 (Roth et al., 2020). In 2022, The American Heart Association included adequate sleep to its cardiovascular checklist “Life's Essential 8”, highlighting the central role of sleep in cardiovascular health (Lloyd‐Jones et al., 2022). Poor sleep quality in healthy subjects is associated with elevated cumulative incidence of cardiovascular outcomes and CVD mortality, even after adjusting for age, sex, smoking status, Body Mass Index (BMI), diabetes mellitus, hypertension, and apnea‐hypopnea index (Yan et al., 2021). Together, available literature points to the critical role of sleep in maintaining optimum cardiovascular health. It is important to note that women are disproportionately at risk of CVD compared to men, such that CVD is the number one cause of mortality in more than 50% of women in the United States (Lloyd‐Jones et al., 2022). However, outside of the increasing focus on menopause, women remain overlooked in prevention strategies targeting CVD, despite emerging risk factors such as posttraumatic stress disorder (PTSD). Of note, premenopausal women are more susceptible to experiencing interpersonal trauma such as rape and sexual assault, and subsequently develop PTSD at a higher rate compared to men (Gatov et al., 2020). Trauma exposure is associated with significant sleep disturbances (Lalley‐Chareczko et al., 2017), including disruptive nocturnal behaviors and nightmares, both hallmark of PTSD (Germain, 2013; Ross et al., 1989). A recent meta‐analytic review by Klam et al. highlighted a pattern of sleep disturbances in PTSD patients (Lam et al., 2023). Specifically, they found that compared to controls, PTSD patients have lower sleep efficiency (SE) and more fragmented sleep. This is consistent with reports of a study comparing women with PTSD to women without PTSD (Calhoun et al., 2007). Thus, trauma exposure could be putting premenopausal women at risk for CVD by virtue of sleep disturbances. Despite this evidence, plausible pathophysiologic pathways between sleep disturbances and cardiovascular biomarkers such as HR and blood pressure (BP) in young trauma‐exposed women are unknown.

Elevated BP and HR can serve as early biomarkers of CVD (Fuchs & Whelton, 2020; Palatini, 2011). High BP is a well‐established risk factor, and elevated resting HR is linked to an increased risk of developing hypertension and CVD events and mortality (Cierpka‐Kmieć & Hering, 2020; Ho et al., 2014; Ma et al., 2022; Tian et al., 2019). The aim of this manuscript was to examine relationships between measures of sleep (objective and subjective) and baseline hemodynamics in a sample of trauma‐exposed women from our ongoing research project examining biomarkers of CVD risk (Tahsin et al., 2024). In this study, we hypothesized that poor sleep (quality and duration) would be associated with higher resting HR and BP, even after controlling for potential covariates (age, BMI, PTSD symptom severity and medications) in premenopausal women with a history of trauma exposure.

## 2 MATERIALS AND METHODS

### 2.1 Ethical overview

All procedures in this study were approved by the Institutional Review Board of the University of Minnesota. During the first in‐person visit, participants signed a written informed consent. This study only included premenopausal women. Therefore, the results may not be generalizable to other populations. However, given that young women are underrepresented in research and are increasingly at risk of trauma exposure and its related complications, including CVD, we believe the value of studying this population outweighs the potential limitations of generalizability.

### 2.2 Study sample

One‐hundred and thirty‐one otherwise healthy premenopausal women (18–49 years) with a history of trauma exposure were included in the study. Women were recruited from the University's campus and the surrounding community, including women's shelters. Participants initially completed a screening survey online or via phone interview; upon completion, those who met the inclusion criteria were invited for a laboratory visit. Eligibility criteria have been previously reported (Ahmed et al., 2024; Corbin et al., 2025; Tahmin et al., 2024; Tahsin et al., 2023; Tahsin et al., 2024). Broadly, participants on antidepressants and anxiolytics were included. Although participants with preexisting CVD and/or on antihypertensive medication were excluded, we included participants who presented with elevated resting BP during the first in‐person visit (*n* = 2). We also excluded participants with a history of substance use in the last 6 months. Participants self‐reported their trauma history in writing as part of a standardized PTSD symptom questionnaire (described later in the *Measures* section). While we encouraged participants to identify the trauma that currently affects them the most, they were also welcome to report additional traumatic experiences if relevant. A subset of the sleep data has been previously published as part of a manuscript looking at SE, arterial stiffness, and microvascular endothelial function (Tahsin et al., 2023). Additionally, we did not capture participants' dietary habits or socioeconomic status.

### 2.3 Experimental design

Eligible participants were invited for in‐person visits, mostly between 8:00 am and 1:00 pm, as previously described (Ahmed et al., 2024; Corbin et al., 2025; Tahmin et al., 2024; Tahsin et al., 2023; Tahsin et al., 2024). During the first in‐person visit, we recorded the participant's anthropometric measurements (height, weight, BMI, and abdominal circumference) and hemodynamic data (resting seated BP and HR). Participants were also asked to complete questionnaires about sleep and mental health. At the end of the visit, the participants were provided with an actigraphy watch to objectively track their sleep/wake cycle for seven consecutive days. The second visit took place during the early follicular phase of their cycle (7 days after the first visit). Participants returned the actigraphy watch and the sleep diary at that visit, and we collected resting supine HR and BP.

### 2.4 Measures

#### 2.4.1 Psychological assessment tools

As previously described (Tahsin et al., 2023; Tahsin et al., 2024), we collected data on the mental health condition of our participants using three self‐reported measures: the *PTSD checklist for DSM‐5* (PCL5), the *Beck Depression Inventory‐II* (*BDI‐II*) *and* the *State*–*Trait Anxiety Inventory* (STAI). For reporting traumatic events and PTSD symptoms severity, we used PCL‐5 with criterion A. The PCL5 is a 20‐item self‐reported questionnaire based on the fifth edition of the Diagnostic and Statistical Manual of Mental Disorders (DSM‐5) symptoms of PTSD, a psychometrically sound measure in individuals at high risk for trauma‐exposure (Morrison et al., 2021; National Center for PTSD, 2024). Participants reported how much they were bothered over the past month by symptoms related to that traumatic event, using a five‐point Likert scale (0=“not at all,” 1 = “a little bit,” 2 = “moderately,” 3 = “Quite a bit,” 4 = “extremely”). The criterion A (i.e., the traumatic event) was used to determine whether the trauma was interpersonal or non‐interpersonal. The total score for PCL5 ranges from 0 to 80 (Blanchard et al., 1996). The BDI‐II is a widely used, 21‐item self‐report questionnaire that measures the severity of depression symptoms in adolescents and adults (Beck et al., 1996). The STAI is a psychological assessment tool used to measure anxiety levels. It consists of two scales: the State Anxiety scale, which measures current feelings, and the Trait Anxiety scale, which measures general anxiety tendencies. The STAI has been widely used in both clinical and research settings to assess anxiety in various populations (Barnes et al., 2002; Ortuño‐Sierra et al., 2016).

#### 2.4.2 Subjective sleep quality

We assessed participants' subjective sleep quality using the *Pittsburgh Sleep Quality Index* (PSQI) and insomnia symptoms using the Insomnia *Severity Index* (ISI). The PSQI consists of a 19‐item self‐rated questionnaire, with 7 components, with scores ranging from 0 to 3 on each component. The global PSQI score ranges from 0 to 21, with higher scores >5 suggesting poor sleep quality (Buysse et al., 1989). The Insomnia Severity Index (ISI) (Bastien et al., 2001) is a 7‐item self‐report measure of insomnia symptoms. Items are rated between 0 (“not at all”) and 4 (“very much”). All items are summed to determine the ISI score (range: 0–28; 0–7 = no insomnia, 8–14 = subthreshold, 15–21 = moderate, and 22–28 = severe insomnia).

#### 2.4.3 Objective sleep quality

We quantified sleep quality using 24‐h wrist‐worn actigraphy. Wrist actigraphy measures rest/activity in 30‐s epochs, which allows inference of 24‐h sleep/wake cycles. We used the Actiwatch Spectrum Plus (Philips Respironics, Bend, OR, USA) with a 32 Hz sampling rate and employed the device's proprietary algorithm (Version 6) to derive discrimination of wake versus sleep. The Actiwatch Spectrum Plus measures ambient illumination and physical activity continuously. Physical activity is measured via a MEMS‐type accelerometer and is expressed in counts per minute, a dimensionless measure of motion that is designed to remove the effects of gravity, transportation, and other types of acceleration that do not indicate subjects' physical activity (Izmailova et al., 2019). Participants wore the Actiwatch for seven consecutive nights and days, which included weekdays and weekends (i.e., Mondayto Sunday). Because of known imprecision of actigraphy in determining the beginning and ending of a sleep period (Blackwell et al., 2005), a sleep diary was kept simultaneously and used to adjudicate the start and the end of the primary sleep period. Data were averaged over the wear period. Our sleep measure of interest was SE, defined as the percentage of time spent asleep while in bed. Additionally, we collected other objective sleep measures such as total sleep duration (TST), sleep latency, and wake time after sleep onset.

#### 2.4.4 Resting blood pressure and heart rate

During Visit 1, when participants received the watch, three seated BP and HR measurements were obtained and averaged using an automated vital signs monitor (Omron Healthcare, Japan). Seven days later, when participants came back to return the watch (Visit 2), we recorded supine HR and BP via peripheral arterial tonometry (EndoPAT, Itamar Medical, Israel) and pulse wave analysis (SphygmoCor XCEL, Atcor Medical, Sydney, Australia) as previously described (Tahsin et al., 2023). All measurements were done at rest.

### 2.5 Data analysis

#### 2.5.1 Analysis of objective sleep data

We used two methods to analyze the actigraphy data: First, we conducted analyses using solely the sleep parameters derived from the actigraphy data (i.e., the software's automatic calculation of awake and asleep times). Second, we used the sleep diary to determine the boundaries of sleep actigraphy data to include in the analyses (sensitivity analyses). Sensitivity analyses were conducted by altering the threshold(s) for concordance between the Actiware software automatic calculation of time spent in bed and participants' reports of time spent in bed in their sleep diary. These analyses take into account the large discrepancy that can occur between the actigraphy and the diary data for the beginning and end of the intended sleep period (Blackwell et al., 2005).

### 2.6 Statistical analysis

All data were analyzed using SPSS 29.0 software (IBM SPSS, NY). All data were tested for normal distribution. We analyzed demographic characteristics using descriptive statistics. Next, to minimize the likelihood of making a type‐II error with multiple testing, bivariate Pearson product correlations were used to identify additional variables (i.e., age, BMI, BP, PSQI, PCL5 and medication use) to be included in a multivariate regression model predicting resting supine hemodynamics (BP and HR) using sleep measures. We ran two regression models. We conducted a stepwise regression (Model 1) which is a method used to build a regression model by iteratively adding or removing predictor variables. It aims to identify the most relevant predictors for a response variable, improving the model's predictive power and simplicity. This process involves evaluating the statistical significance of each predictor at each step and either including it in the model (if significant) or removing it (if not significant). All data are reported as mean ± SD. In the second regression (Model 2), we included all identified covariates (i.e., age, BMI, BP, PSQI, PCL5, and medication use).

## 3 RESULTS

Table 1 shows baseline descriptive characteristics of all 131 women included in our study. Our participants were predominantly white (70%), on average 26 ± 7 years old with a BMI of 26.4 ± 6.1 kg/m^2^ and normotensive. The majority (78.5%) of our participants reported interpersonal trauma, presented with poor sleep (PSQI >5, SE <85%), moderate depression, moderate anxiety, moderate PTSD symptoms severity, and were mainly on antidepressants (49%).

**TABLE 1:** Descriptive characteristics of participants.

Variables	Mean	Std. deviation	Min–max
Age (years)	26	7	18–49
BMI (kg/m^2^)	26.4	6.1	12.3–43.5
Race^a^			
White (*n*) %	(91) 70%		
African‐American (*n*) %	(22) 16.9%		
Hispanic (*n*) %	(6) 4.6%		
Asian (*n*) %	(10) 7.7%		
Native American (*n*) %	(1) 0.7%		
Hemodynamic measures			
Seated systolic BP (mmHg)	107	10	80–137
Seated diastolic BP (mmHg)	69	9	48–100
Seated HR (beats/min)	77	13	52–125
Respiratory rate (breaths/min)	14	3	7–24
Trauma History			
Interpersonal (*n*) %	(102) 78.5%		
Non‐interpersonal (*n*) %	(28) 21.5%		
Sleep and mental health questionnaires			
PSQI (a.u.)	9	3	2–19
ISI (a.u.)	12.0	6.0	0–26
PCL5 (a.u.)	36	16	0–78
BDI (a.u.)	19	11	0–55
STAI‐Trait (a.u.)	45	12	20–70
STAI‐State (a.u.)	48	13	6–80
Medications			
Antidepressants (*n*) %	(68) 48.9%		
Anxiolytics (*n*) %	(8) 5.8%		
Sleep (*n*) %	(9) 6.5%		
Objective sleep measures			
Sleep efficiency (%)	81.8	5.6	59.17–92.26
Sleep latency (min)	29.2	20.2	0.5–127.4
WASO (min)	45.7	16.8	16.92–106
Total sleep time (min)	434.3	51.6	305.4–614
Total activity (count/day)	219689.3	64084.3	76,786–407849.6

*Note*: Values are means ± SD unless otherwise specified, minimums, and maximums; *n* = 131 participants.

Abbreviations: BDI, Beck's depression inventory; BMI, Body Mass Index; BP, blood pressure; HR, heart rate; ISI, Insomnia Severity Index; PCL5, posttraumatic stress disorder checklist for DSM‐5 criterion A; PSQI, Pittsburgh sleep quality index; STAI, State–trait anxiety inventory; WASO, Wake time after sleep onset.

^a^ One missing data point for Race.

Table 2 shows the correlations between dependent variables, independent variables, and all potential predictors. Supine HR was correlated with age (*r* = −0.248, *p* = 0.004), supine diastolic BP (*r* = 0.322, *p* < 0.001) and SE (*r* = −0.222, *p* = 0.010). Supine diastolic BP was correlated with age (*r* = 0.241, *p* = 0.005), PCL5 (*r* = 0.230, *p* = 0.008) and ISI (*r* = 0.192, *p* = 0.026). Supine systolic BP was correlated with age (*r* = 0.303, *p* < 0.001) and BMI (*r* = 0.368, *p* < 0.001) and showed no correlation with either sleep measures.

**TABLE 2:** Correlation matrix with all variables of interest.

	Age	BMI	PSQI	ISI	PCL5	Supine SBP	Supine DBP	Supine HR	SE	TST	WASO	Latency
Age	‐											
BMI	0.194*	‐										
PSQI	−0.024	0.117	‐									
ISI	0.027	0.197*	0.748**	‐								
PCL5	0.100	0.134	0.358**	0.410**	‐							
Supine SBP	0.303**	0.368**	0.099	0.158	0.130	‐						
Supine DBP	0.241**	0.163	0.157	0.192*	0.230**	0.713**	‐					
Supine HR	−0.248**	0.116	0.140	0.136	0.118	0.157	0.322**	‐				
SE	0.032	0.101	−0.127	−0.107	−0.050	0.093	−0.015	−0.222**	‐			
TST	0.038	0.023	0.023	0.018	−0.042	0.085	0.048	−0.041	0.317**	‐		
WASO	0.087	−0.102	0.207*	0.223**	0.012	−0.041	0.100	0.082	−0.547**	0.133	‐	
Latency	−0.104	−0.001	0.076	0.098	0.052	−0.164	−0.111	0.123	−0.588**	0.045	0.108	‐

*Note*: Significance values are two‐tailed.

Abbreviations: BMI, Body Mass Index; DBP, diastolic blood pressure; HR, heart rate; ISI, Insomnia Severity Index; PCL5, posttraumatic stress disorder checklist for DSM‐5 criterion A; PSQI, pittsburgh sleep quality index; SBP, systolic blood pressure; SE, sleep efficiency; TST, total sleep time; WASO, wake time after sleep onset.

* *p* < 0.05.

** *p* < 0.01.

**FIGURE 1:**
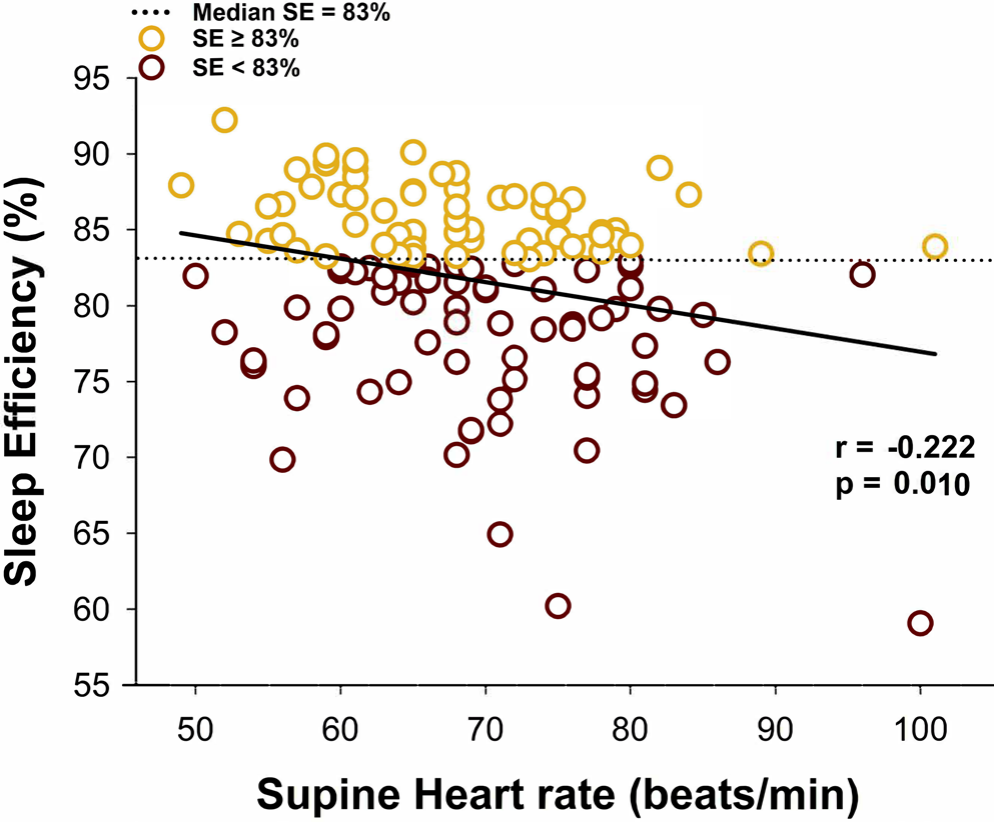
The association between resting heart rate (HR; beats/min) and sleep efficiency (SE; %) in 131 young, healthy, premenopausal trauma‐exposed women. Pearson product correlation coefficient was used to assess the relationship, and a *p* <0.05 was considered as significant. Resting heart rate was negatively associated with sleep efficiency in our sample (*r* = −0.222, *p* = 0.010).

Figure 1 shows the scatterplot depicting the bivariate correlation between SE (%) and resting HR (beats/min) in all women. The median SE for our sample (83%) is represented by a dashed line, while the solid line is the line of best fit. Gold circles represent participants with SE equal to or above the sample median (SE ≥83%) and maroon circles those with SE below the sample median (SE <83%). Overall, SE was negatively correlated to HR in our sample. The weaker correlation between ISI and supine diastolic BP was not graphed. No other significant relationships were found between sleep variables and baseline hemodynamics, as seen in Table 2. These results remain consistent with our sensitivity analyses.

Table 3 highlights the results of the regression analyses predicting supine HR. Given the association between SE and HR, we conducted two multivariate regression models investigating the effect of sleep quality (Actiwatch derived SE) on supine resting HR (dependent variable) while accounting for the potential influence of age, BMI, BP, PTSD symptom severity (PCL5), and medications (antidepressants, anxiolytics and sleep‐aid) on the relationship. The final model of the stepwise regression (Model 1) only included age (*β* = −0.328, *p* < 0.001), diastolic BP (*β* = 0.390, *p* < 0.001), and SE (*β* = −0.206, *p* = 0.008), the three identified significant predictors. This model accounted for 25% of the variance in resting HR in this population of trauma‐exposed young women (*R* ^2^ = 0.253, *p* = 0.008). Of note, variables not retained in the final model were neither significant predictors nor did they improve the model. The model with all identified covariates included (Model 2) just slightly increased the variance explained (*R* ^2^ = 0.287, *p* < 0.001). We also conducted a multiple linear regression model predicting supine diastolic BP. Unlike SE, ISI (*β* = −0.051, *p* = 0.531) was not a significant predictor of diastolic BP after controlling for all covariates.

**TABLE 3:** Multivariate regression model for supine resting heart rate.

Variables	Model 1		Model 2	
	β‐coefficient	95% CI	β‐coefficient	95% CI
(Constant)		99.62, 171.93		53.15, 107.10
Age (years)	−0.328**	−0.700, −0.249	−0.356**	−0.743, −0.287
Supine DBP (mmHg)	0.390**	0.289, 0.675	0.351**	0.235, 0.634
Sleep efficiency (%)	−0.206**	−0.617, −0.094	−0.228**	−0.660, −0.127
BMI (kg/m^2^)			0.130	−0.044, 0.466
PCL5			0.034	−0.074, 0.113
Depression Rx			0.055	−2.072, 4.223
Anxiety Rx			0.015	−5.617, 6.870
Sleep aid			0.103	−1.962, 9.967
*R* ^2^	**0.253**		**0.287**	
*p*‐value	0.008		<0.001	

*Note*: *n* = 131 participants.

Abbreviations: BMI, Body Mass Index; DBP, diastolic; PCL5, posttraumatic stress disorder checklist for DSM‐5 criterion A; Rx, medication. R^2^ values are bolded.

** *p* < 0.01.

## 4 DISCUSSION

In this study, we examined the association between sleep (quality and duration) and resting hemodynamics (HR and BP), while accounting for various covariates in a group of trauma‐exposed young women (predominantly interpersonal trauma). Our main finding is that objective SE measured via Actiwatch is negatively associated with supine HR in this population even after controlling for age, BMI, diastolic BP, PTSD symptom severity, and medications for depression, anxiety, and sleep. We also found a weak correlation between ISI and diastolic BP, highlighting the possible effect of insomnia symptoms on resting BP. These findings in trauma‐exposed women could represent an important physiological substrate that could serve to identify individuals, particularly premenopausal women, at higher risk of developing mental health‐associated CVD.

Elevated BP and HR can serve as early indicators of CVD. High BP is a well‐established risk factor, and elevated resting HR (e.g., consistently above 80 bpm) is linked to an increased risk of developing hypertension, coronary heart disease, heart failure, stroke, and overall mortality (Cierpka‐Kmieć & Hering, 2020; Ho et al., 2014; Ma et al., 2022; Tian et al., 2019). Multiple large epidemiological studies have consistently shown that elevated resting HR is associated with increased risks of coronary heart disease, sudden cardiac death, and overall cardiovascular mortality, even after adjusting for other traditional risk factors (Fox et al., 2007). Moreover, recent analyses using the Sleep Heart Health Study data revealed that poor SE was significantly associated with primary and secondary composite cardiovascular outcomes (Yan et al., 2021). SE was found to be a predictor of CVD mortality (HR, 1.887; 95% CI, 1.224–2.909; *p* = 0.004) (Yan et al., 2021). Although the literature provides reports of an association between poor sleep quality and elevated HR, those prior studies either relied only on subjective measures of sleep (Sajjadieh et al., 2020) or were conducted in a disease population (i.e., sleep disorders) (Andreas et al., 1992). Therefore, in the current study, we also collected objective sleep parameters via actigraphy in an otherwise healthy population of women. We found that lower SE was associated with increased HR in trauma‐exposed premenopausal women. Relative to the trauma‐exposed nature of our population, psychiatric disorders such as PTSD and anxiety that are often associated with higher resting HR (Latvala et al., 2016; Sadeghi et al., 2022) have been linked to premature development of CVD (Boscarino, 2008; Roest et al., 2010). A 30‐year follow‐up study of a Swedish cohort found that patients with psychiatric disorders experienced higher rates of CVD in the first and subsequent years of diagnosis compared to unaffected twin siblings (Shen et al., 2023). Further, it appears that the association between PTSD and elevated resting HR might be bidirectional and related to the severity of PTSD and/or specific symptoms. Recent studies have revealed that elevated HR after trauma exposure is associated with the development of PTSD 4 months after the traumatic incident (Morris et al., 2016; Shalev et al., 1998). Fonkoue et al. reported higher resting HR in veterans with severe PTSD symptoms (Fonkoue, Marvar, et al., 2020), while Sadeghi et al. found a link between elevated resting HR and hyperarousal symptoms (Sadeghi et al., 2022).

Poor sleep quality and insufficient sleep duration can also negatively impact resting blood pressure, potentially leading to hypertension. Specifically, shorter sleep duration and poorer sleep quality are associated with higher systolic and diastolic blood pressure readings (Ross et al., 2014). This relationship is likely due to the role of sleep in the regulation of hormones that control stress and metabolism, which directly affect BP. Adequate sleep is crucial for maintaining cardiovascular function by allowing BP to dip at night. Disrupted or poor sleep quality prevents this nocturnal dip because of the overactivity of the sympathetic nervous system (Huart et al., 2023). Experimental studies using different sleep deprivation models have shown that inadequate sleep is associated with an increase in sympathetic activity, BP, and HR (Tai et al., 2023; Sauvet et al., 2010). Further, in premenopausal trauma‐exposed women, Tahsin et al. reported that SE is a strong predictor of arterial stiffness, which may indirectly lead to an overactive sympathetic activity and subsequently to changes in BP via decreased sensitivity of baroreceptors located in the vascular wall (Tahsin et al., 2023). In our current study, we found a weak positive association between subjective poor sleep as measured via ISI and diastolic BP. It is possible that hemodynamic changes in our population are still in the early stages and have not yet affected systolic BP. Regarding sleep duration, experimental data suggest that reducing sleep length may result in adverse responses such as an increase in HR (Baharav et al., 1995). Kuciene and Dulskiene (Kuciene & Dulskiene, 2014) found that short sleep duration was associated with higher BP, while other studies reported no association (Shaikh et al., 2010) between sleep duration and BP. Consistent with this study by Shaik et al. (Shaikh et al., 2010), we did not find any associations between TST or WASO and resting hemodynamics (BP and HR) in our population of trauma‐exposed women.

Although not the primary objective of our manuscript, we found minimal to no correlation between objective sleep measures via actigraphy and subjective sleep quality assessed via PSQI. Wake after sleep onset was the only measure to be mildly correlated with PSQI. However, our result is consistent with the findings of Calhoun et al. (Calhoun et al., 2007) in a study of 30 women with PTSD and 22 women without PTSD. They reported that three nights of actigraphy indicated poorer SE, increased sleep latency, and more restless sleep in women with PTSD; but these measures were unrelated to scores on the PSQI. Likewise, a study (Aili et al., 2017) in 54 adults where sleep was assessed for 7 consecutive nights using actigraphy as an objective measure, and the Karolinska sleep diary for a subjective measure of quality, reported a low correlation between the investigated actigraphy sleep parameters and subjective sleep quality. These aforementioned studies suggest that the two methods of sleep assessment may capture different dimensions of sleep.

Age and BMI are known to be independent predictors of CVD (Khan et al., 2018). Patients with psychiatric disorders often present with higher BMI due to associated behavioral risk factors and comorbidities like eating disorders (Da Luz et al., 2018; Magallares & Pais‐Ribeiro, 2014). In our model, age and not BMI was a strong predictor of HR. Prior work from our laboratory revealed that age and SE are strong predictors of arterial stiffness in premenopausal trauma‐exposed women (Tahsin et al., 2023). Additionally, almost half of our sample in the current study was on antidepressants, but our results remained consistent even after we controlled for anxiety, depression, and sleep medications. Sleep disturbances such as frequent nighttime awakenings and insomnia are hallmarks of PTSD (Germain, 2013; Ross et al., 1989); and other psychiatric disorders such as anxiety and depression (Freeman et al., 2020). These conditions are often comorbid in individuals exposed to trauma, and their association with CVD is most notable among younger women and may be mediated by stress‐related neuro‐immune pathways (Civieri et al., 2024).

There are important limitations of this study. First, our study did not include the gold‐standard sleep measure, polysomnography, which can provide precision in cardiovascular measurements by sleep stages, which modulate HR, BP, and overall autonomic nervous system activity during the night. Second, our study was cross‐sectional; thus, limiting directionality in the relationship between HR and SE. Third, we recruited women from various socioeconomic statuses and housing situations (such as shelters) and could not account for those variables in our analyses because the data was not systematically recorded. Fourth, the current study did not evaluate trauma load, the timeline of trauma, or how associations may differ by event type. Fifth, we did not collect nighttime and ambulatory BP and HR; therefore, we could not look at the effect of sleep on hemodynamics outside of the laboratory. Sixth, we excluded from this study trans and non‐binary individuals, who often experience high rates of trauma. However, since young women are underrepresented in research and are increasingly at risk for PTSD and its complications, studying this population is valuable despite the limitations in generalizability.

## 5 CONCLUSION

Women, outside of menopause, remain overlooked in prevention strategies targeting CVD, the leading cause of death in women. Trauma and subsequent development of PTSD are emerging risk factors for CVD in women, by virtue of sleep disturbances. Given that elevated BP and HR serve as early biomarkers of CVD, the aim of this manuscript was to examine relationships between sleep measures and baseline hemodynamics in a sample of trauma‐exposed women. Findings from the current study have important clinical implications. We found that SE in trauma‐exposed women strongly predicts resting heart rate, even when controlling for age, BMI, diastolic BP, PTSD symptom severity, and medications for depression, anxiety, and sleep. We also found a weak association between ISI and diastolic BP. This indicates that in this trauma‐exposed premenopausal population of women, poor sleep could be a screening tool to detect women at risk for future development of CVD that may benefit from early targeted sleep interventions. These findings could serve as initial evidence to support future inquiry into this approach.

## FUNDING INFORMATION

This study was supported by the following grants: K01HL161027 (NHLBI) and UMN CTSI UL1TR002494.

## CONFLICTS OF INTEREST

The authors declare no competing interests.

## Data Availability

The data included in this manuscript will be available upon request.

## References

[phy270525-bib-0001] Ahmed, Z. , Tahmin, C. I. , Tahsin, C. T. , Michopoulos, V. , Mohamed, A. , Wattero, R. , Albott, S. , Cullen, K. R. , Lowe, D. A. , Osborn, J. , & Fonkoue, I. T. (2024). Higher arterial stiffness and blunted vagal control of the heart in young women with compared to without a clinical diagnosis of PTSD. Clinical Autonomic Research, 34, 165–175. 38324188 10.1007/s10286-024-01014-7 PMC10947824

[phy270525-bib-0002] Aili, K. , Åström‐Paulsson, S. , Stoetzer, U. , Svartengren, M. , & Hillert, L. (2017). Reliability of actigraphy and subjective sleep measurements in adults: The design of sleep assessments. Journal of Clinical Sleep Medicine, 13, 39–47. 27707448 10.5664/jcsm.6384 PMC5181612

[phy270525-bib-0003] Andreas, S. , Hajak, G. , von Breska, B. , Rüther, E. , & Kreuzer, H. (1992). Changes in heart rate during obstructive sleep apnoea. The European Respiratory Journal, 5, 853–857. 1499710

[phy270525-bib-0004] Baharav, A. , Kotagal, S. , Gibbons, V. , Rubin, B. K. , Pratt, G. , Karin, J. , & Akselrod, S. (1995). Fluctuations in autonomic nervous activity during sleep displayed by power spectrum analysis of heart rate variability. Neurology, 45, 1183–1187. 7783886 10.1212/wnl.45.6.1183

[phy270525-bib-0005] Barnes, L. L. B. , Harp, D. , & Jung, W. S. (2002). Reliability generalization of scores on the Spielberger state‐trait anxiety inventory. Educational and Psychological Measurement, 62, 603–618.

[phy270525-bib-0006] Bastien, C. H. , Vallières, A. , & Morin, C. M. (2001). Validation of the insomnia severity index as an outcome measure for insomnia research. Sleep Medicine, 2, 297–307. 11438246 10.1016/s1389-9457(00)00065-4

[phy270525-bib-0007] Beck, A. T. , Steer, R. A. , & Brown, G. K. (1996). Manual for the beck depression inventory‐II. Psychological Corporation.

[phy270525-bib-0008] Blackwell, T. , Ancoli‐Israel, S. , Gehrman, P. R. , Schneider, J. L. , Pedula, K. L. , & Stone, K. L. (2005). Actigraphy scoring reliability in the study of osteoporotic fractures. Sleep, 28, 1599–1605. 16408420 10.1093/sleep/28.12.1599

[phy270525-bib-0009] Blanchard, E. B. , Jones‐Alexander, J. , Buckley, T. C. , & Forneris, C. A. (1996). Psychometric properties of the PTSD checklist (PCL). Behaviour Research and Therapy, 34, 669–673. 8870294 10.1016/0005-7967(96)00033-2

[phy270525-bib-0010] Boscarino, J. A. (2008). A prospective study of PTSD and early‐age heart disease mortality among Vietnam veterans: Implications for surveillance and prevention. Psychosomatic Medicine, 70, 668–676. 18596248 10.1097/PSY.0b013e31817bccaf PMC3552245

[phy270525-bib-0011] Buysse, D. J. , Reynolds, C. F., 3rd , Monk, T. H. , Berman, S. R. , & Kupfer, D. J. (1989). The Pittsburgh sleep quality index: A new instrument for psychiatric practice and research. Psychiatry Research, 28, 193–213. 2748771 10.1016/0165-1781(89)90047-4

[phy270525-bib-0012] Calhoun, P. S. , Wiley, M. , Dennis, M. F. , Means, M. K. , Edinger, J. D. , & Beckham, J. C. (2007). Objective evidence of sleep disturbance in women with posttraumatic stress disorder. Journal of Traumatic Stress, 20, 1009–1018. 18157880 10.1002/jts.20255

[phy270525-bib-0013] Cierpka‐Kmieć, K. , & Hering, D. (2020). Tachycardia: The hidden cardiovascular risk factor in uncomplicated arterial hypertension. Cardiology Journal, 27, 857–867. 30799548 10.5603/CJ.a2019.0021 PMC8079088

[phy270525-bib-0014] Civieri, G. , Abohashem, S. , Grewal, S. S. , Aldosoky, W. , Qamar, I. , Hanlon, E. , Choi, K. W. , Shin, L. M. , Rosovsky, R. P. , Bollepalli, S. C. , Lau, H. C. , Armoundas, A. , Seligowski, A. V. , Turgeon, S. M. , Pitman, R. K. , Tona, F. , Wasfy, J. H. , Smoller, J. W. , Iliceto, S. , … Tawakol, A. (2024). Anxiety and depression associated with increased cardiovascular disease risk through accelerated development of risk factors. JACC. Advances, 3, 101208. 39238850 10.1016/j.jacadv.2024.101208 PMC11375258

[phy270525-bib-0015] Corbin, C. , Tahmin, C. I. , Tahsin, C. T. , Ahmed, Z. , Wattero, R. , Mohamed, A. , Racette, S. B. , Duprez, D. , & Fonkoue, I. T. (2025). Estradiol levels are differentially associated with pulse wave velocity in trauma‐exposed premenopausal women with and without PTSD. American Journal of Physiology. Regulatory, Integrative and Comparative Physiology, 328, R235–R241. 39824513 10.1152/ajpregu.00262.2024 PMC12167166

[phy270525-bib-0016] Da Luz, F. Q. , Hay, P. , Touyz, S. , & Sainsbury, A. (2018). Obesity with comorbid eating disorders: Associated health risks and treatment approaches. Nutrients, 10, 829. 29954056 10.3390/nu10070829 PMC6073367

[phy270525-bib-0017] Fonkoue, I. T. , Marvar, P. J. , Norrholm, S. , Li, Y. , Kankam, M. L. , Jones, T. N. , Vemulapalli, M. , Rothbaum, B. , Bremner, J. D. , Le, N.‐A. , & Park, J. (2020). Symptom severity impacts sympathetic dysregulation and inflammation in post‐traumatic stress disorder (PTSD). Brain, Behavior, and Immunity, 83, 260–269. 31682970 10.1016/j.bbi.2019.10.021 PMC6906238

[phy270525-bib-0019] Fox, K. , Borer, J. S. , Camm, A. J. , Danchin, N. , Ferrari, R. , Lopez Sendon, J. L. , Steg, P. G. , Tardif, J.‐C. , Tavazzi, L. , Tendera, M. , & Heart Rate Working Group . (2007). Resting heart rate in cardiovascular disease. Journal of the American College of Cardiology, 50, 823–830. 17719466 10.1016/j.jacc.2007.04.079

[phy270525-bib-0020] Freeman, D. , Sheaves, B. , Waite, F. , Harvey, A. G. , & Harrison, P. J. (2020). Sleep disturbance and psychiatric disorders. Lancet Psychiatry, 7, 628–637. 32563308 10.1016/S2215-0366(20)30136-X

[phy270525-bib-0021] Fuchs, F. D. , & Whelton, P. K. (2020). High blood pressure and cardiovascular disease. Hypertension, 75, 285–292. 31865786 10.1161/HYPERTENSIONAHA.119.14240 PMC10243231

[phy270525-bib-0022] Gatov, E. , Koziel, N. , Kurdyak, P. , Saunders, N. R. , Chiu, M. , Lebenbaum, M. , Chen, S. , & Vigod, S. N. (2020). Epidemiology of interpersonal trauma among women and men psychiatric inpatients: A population‐based study. Canadian Journal of Psychiatry, 65, 124–135. 31262196 10.1177/0706743719861374 PMC6997970

[phy270525-bib-0023] Germain, A. (2013). Sleep disturbances as the hallmark of PTSD: Where are we now? The American Journal of Psychiatry, 170, 372–382. 23223954 10.1176/appi.ajp.2012.12040432 PMC4197954

[phy270525-bib-0024] Ho, J. E. , Larson, M. G. , Ghorbani, A. , Cheng, S. , Coglianese, E. E. , Vasan, R. S. , & Wang, T. J. (2014). Long‐ term cardiovascular risks associated with an elevated heart rate: The Framingham heart study. Journal of the American Heart Association, 3, e000668. 24811610 10.1161/JAHA.113.000668 PMC4309047

[phy270525-bib-0025] Huart, J. , Persu, A. , Lengelé, J.‐P. , Krzesinski, J.‐M. , Jouret, F. , & Stergiou, G. S. (2023). Pathophysiology of the nondipping blood pressure pattern. Hypertension, 80, 719–729. 36606502 10.1161/HYPERTENSIONAHA.122.19996

[phy270525-bib-0026] Izmailova, E. S. , McLean, I. L. , Hather, G. , Merberg, D. , Homsy, J. , Cantor, M. , Volfson, D. , Bhatia, G. , Perakslis, E. D. , Benko, C. , & Wagner, J. A. (2019). Continuous monitoring using a wearable device detects activity‐induced heart rate changes after administration of amphetamine. Clinical and Translational Science, 12, 677–686. 31365190 10.1111/cts.12673 PMC6853263

[phy270525-bib-0027] Khan, S. S. , Ning, H. , Wilkins, J. T. , Allen, N. , Carnethon, M. , Berry, J. D. , Sweis, R. N. , & Lloyd‐Jones, D. M. (2018). Association of body mass index with lifetime risk of cardiovascular disease and compression of morbidity. JAMA Cardiology, 3, 280–287. 29490333 10.1001/jamacardio.2018.0022 PMC5875319

[phy270525-bib-0028] Kuciene, R. , & Dulskiene, V. (2014). Associations of short sleep duration with prehypertension and hypertension among Lithuanian children and adolescents: A cross‐sectional study. BMC Public Health, 14, 255. 24628980 10.1186/1471-2458-14-255 PMC3984754

[phy270525-bib-0029] Lalley‐Chareczko, L. , Segal, A. , Perlis, M. L. , Nowakowski, S. , Tal, J. Z. , & Grandner, M. A. (2017). Sleep disturbance partially mediates the relationship between intimate partner violence and physical/mental health in women and men. Journal of Interpersonal Violence, 32, 2471–2495. 26149676 10.1177/0886260515592651 PMC4710553

[phy270525-bib-0030] Lam, L. , Ho, F. Y.‐Y. , Wong, V. W.‐H. , Chan, K.‐W. , Poon, C.‐Y. , Yeung, W.‐F. , & Chung, K.‐F. (2023). Actigraphic sleep monitoring in patients with post‐traumatic stress disorder (PTSD): A meta‐analysis. Journal of Affective Disorders, 320, 450–460. 36174789 10.1016/j.jad.2022.09.045

[phy270525-bib-0031] Latvala, A. , Kuja‐Halkola, R. , Rück, C. , D'Onofrio, B. M. , Jernberg, T. , Almqvist, C. , Mataix‐Cols, D. , Larsson, H. , & Lichtenstein, P. (2016). Association of resting heart rate and blood pressure in late adolescence with subsequent mental disorders: A longitudinal population study of more than 1 million men in Sweden. JAMA Psychiatry, 73, 1268–1275. 27784035 10.1001/jamapsychiatry.2016.2717

[phy270525-bib-0032] Lloyd‐Jones, D. M. , Allen, N. B. , Anderson, C. A. M. , Black, T. , Brewer, L. C. , Foraker, R. E. , Grandner, M. A. , Lavretsky, H. , Perak, A. M. , Sharma, G. , Rosamond, W. , & American Heart Association . (2022). Life's essential 8: Updating and enhancing the American Heart Association's construct of cardiovascular health: A presidential advisory from the American Heart Association. Circulation, 146, e18–e43. 35766027 10.1161/CIR.0000000000001078 PMC10503546

[phy270525-bib-0033] Ma, R. , Gao, J. , Mao, S. , & Wang, Z. (2022). Association between heart rate and cardiovascular death in patients with coronary heart disease: A NHANES‐based cohort study. Clinical Cardiology, 45, 574–582. 35352385 10.1002/clc.23818 PMC9045079

[phy270525-bib-0034] Magallares, A. , & Pais‐Ribeiro, J. L. (2014). Mental health and obesity: A meta‐analysis. Applied Research in Quality of Life, 9, 295–308.

[phy270525-bib-0035] Morris, M. C. , Hellman, N. , Abelson, J. L. , & Rao, U. (2016). Cortisol, heart rate, and blood pressure as early markers of PTSD risk: A systematic review and meta‐analysis. Clinical Psychology Review, 49, 79–91. 27623149 10.1016/j.cpr.2016.09.001 PMC5079809

[phy270525-bib-0036] Morrison, K. , Su, S. , Keck, M. , & Beidel, D. C. (2021). Psychometric properties of the PCL‐5 in a sample of first responders. Journal of Anxiety Disorders, 77, 102339. 33249315 10.1016/j.janxdis.2020.102339

[phy270525-bib-0037] National Center for PTSD . (2024). PTSD: National center for PTSD. https://www.ptsd.va.gov/

[phy270525-bib-0038] Ortuño‐Sierra, J. , García‐Velasco, L. , Inchausti, F. , Debbané, M. , & Fonseca‐Pedrero, E. (2016). New approaches on the study of the psychometric properties of the STAI. Actas Españolas de Psiquiatría, 44, 83–92. 27254400

[phy270525-bib-0039] Palatini, P. (2011). Role of elevated heart rate in the development of cardiovascular disease in hypertension. Hypertension, 58, 745–750. 21896939 10.1161/HYPERTENSIONAHA.111.173104

[phy270525-bib-0040] Roest, A. M. , Martens, E. J. , de Jonge, P. , & Denollet, J. (2010). Anxiety and risk of incident coronary heart disease: A meta‐analysis. Journal of the American College of Cardiology, 56, 38–46. 20620715 10.1016/j.jacc.2010.03.034

[phy270525-bib-0041] Ross, A. J. , Yang, H. , Larson, R. A. , & Carter, J. R. (2014). Sleep efficiency and nocturnal hemodynamic dipping in young, normotensive adults. American Journal of Physiology. Regulatory, Integrative and Comparative Physiology, 307, R888–R892. 25031228 10.1152/ajpregu.00211.2014

[phy270525-bib-0042] Ross, R. J. , Ball, W. A. , Sullivan, K. A. , & Caroff, S. N. (1989). Sleep disturbance as the hallmark of posttraumatic stress disorder. The American Journal of Psychiatry, 146, 697–707. 2658624 10.1176/ajp.146.6.697

[phy270525-bib-0043] Roth, G. A. , Mensah, G. A. , Co, J. , Addolorato, G. , Ammirati, E. , Baddour, L. M. , & Global Burden of Cardiovascular Diseases Writing Group . (2020). Global burden of cardiovascular diseases and risk factors, 1990–2019: Update from the GBD 2019 study. Journal of the American College of Cardiology, 76, 2982–3021. 33309175 10.1016/j.jacc.2020.11.010 PMC7755038

[phy270525-bib-0044] Sadeghi, M. , Sasangohar, F. , McDonald, A. D. , & Hegde, S. (2022). Understanding heart rate reactions to post‐traumatic stress disorder (PTSD) among veterans: A naturalistic study. Human Factors, 64, 173–187. 34292055 10.1177/00187208211034024

[phy270525-bib-0045] Sajjadieh, A. , Shahsavari, A. , Safaei, A. , Penzel, T. , Schoebel, C. , Fietze, I. , Mozafarian, N. , Amra, B. , & Kelishadi, R. (2020). The association of sleep duration and quality with heart rate variability and blood pressure. Tanaffos, 19, 135–143. 33262801 PMC7680518

[phy270525-bib-0046] Sauvet, F. , Leftheriotis, G. , Gomez‐Merino, D. , Langrume, C. , Drogou, C. , Van Beers, P. , Bourrilhon, C. , Florence, G. , & Chennaoui, M. (2010). Effect of acute sleep deprivation on vascular function in healthy subjects. Journal of Applied Physiology, 108, 68–75. 19910332 10.1152/japplphysiol.00851.2009

[phy270525-bib-0047] Shaikh, W. A. , Patel, M. , & Singh, S. (2010). Association of sleep duration with arterial blood pressure profile of gujarati Indian adolescents. Indian Journal of Community Medicine, 35, 125–129. 20606936 10.4103/0970-0218.62571 PMC2888340

[phy270525-bib-0048] Shalev, A. Y. , Sahar, T. , Freedman, S. , Peri, T. , Glick, N. , Brandes, D. , Orr, S. P. , & Pitman, R. K. (1998). A prospective study of heart rate response following trauma and the subsequent development of posttraumatic stress disorder. Archives of General Psychiatry, 55, 553–559. 9633675 10.1001/archpsyc.55.6.553

[phy270525-bib-0049] Shen, Q. , Mikkelsen, D. H. , Luitva, L. B. , Song, H. , Kasela, S. , Aspelund, T. , Bergstedt, J. , Lu, Y. , Sullivan, P. F. , Ye, W. , Fall, K. , Tornvall, P. , Pawitan, Y. , Andreassen, O. A. , Buil, A. , Milani, L. , Fang, F. , & Valdimarsdóttir, U. (2023). Psychiatric disorders and subsequent risk of cardiovascular disease: A longitudinal matched cohort study across three countries. EClinicalMedicine, 61, 102063. 37425374 10.1016/j.eclinm.2023.102063 PMC10329128

[phy270525-bib-0050] Tahmin, C. I. , Tahsin, C. T. , Wattero, R. , Ahmed, Z. , Corbin, C. , Carter, J. R. , Park, J. , Racette, S. B. , Sullivan, S. S. , Herr, M. D. , & Fonkoue, I. T. (2024). Blunted brachial blood flow velocity response to acute mental stress in PTSD females. Physiological Reports, 12, e16137. 38969625 10.14814/phy2.16137 PMC11226346

[phy270525-bib-0051] Tahsin, C. T. , Ahmed, Z. , Mohamed, A. , Tahmin, C. I. , Wattero, R. , Corbin, C. , & Fonkoue, I. T. (2024). Psychiatric disorders endorsed by trauma‐exposed premenopausal women enrolled in a cardiovascular research study: A 2‐year report. Discover Mental Health, 4, 52. 39516389 10.1007/s44192-024-00108-y PMC11549263

[phy270525-bib-0052] Tahsin, C. T. , Michopoulos, V. , Powers, A. , Park, J. , Ahmed, Z. , Cullen, K. , Jenkins, N. D. M. , Keller‐Ross, M. , & Fonkoue, I. T. (2023). Sleep efficiency and PTSD symptom severity predict microvascular endothelial function and arterial stiffness in young, trauma‐exposed women. American Journal of Physiology. Heart and Circulatory Physiology, 325, H739–H750. 37505472 10.1152/ajpheart.00169.2023 PMC10642999

[phy270525-bib-0053] Tai, B. W. S. , Dawood, T. , Macefield, V. G. , & Yiallourou, S. R. (2023). The association between sleep duration and muscle sympathetic nerve activity. Clinical Autonomic Research, 33, 647–657. 37543558 10.1007/s10286-023-00965-7 PMC10751264

[phy270525-bib-0054] Tian, J. , Yuan, Y. , Shen, M. , Zhang, X. , He, M. , Guo, H. , Yang, H. , & Wu, T. (2019). Association of resting heart rate and its change with incident cardiovascular events in the middle‐aged and older Chinese. Scientific Reports, 9, 6556. 31024039 10.1038/s41598-019-43045-5 PMC6484081

[phy270525-bib-0055] Yan, B. , Yang, J. , Zhao, B. , Fan, Y. , Wang, W. , & Ma, X. (2021). Objective sleep efficiency predicts cardiovascular disease in a community population: The sleep heart health study. Journal of the American Heart Association, 10, e016201. 33719504 10.1161/JAHA.120.016201 PMC8174351

